# MR-augmented cardio pulmonary exercise testing: an integrated approach to assessment of children with pulmonary hypertension

**DOI:** 10.1186/1532-429X-18-S1-P178

**Published:** 2016-01-27

**Authors:** Nathaniel J Barber, Vivek Muthurangu

**Affiliations:** Institute of Cardiovascular Science, UCL, London, United Kingdom

## Background

Pulmonary arterial hypertension (PAH) is a life limiting disorder resulting in progressive right heart (RV) failure. Cardiac MRI is the reference standard for RV assessment in PH and has known prognostic value in children. Symptoms of PAH are exacerbated or become apparent with exercise and cardiopulmonary exercise testing (CPET) is also prognostic in PAH.

We have recently demonstrated the feasibility of MR augmented CPET (MR-CPET) in healthy adults. Using the Fick principle MR-CPET allows calculation of tissue oxygen extraction ΔcO_2_. A combined approach using MR-CPET could provide a more sensitive method of assessing disease severity, the causes of exercise limitation and response to treatment. In this study we assessed the utility of MR-CPET in children with PAH.

## Methods

10 Children with PAH aged 8 to 16 years (WHO class I or II, on oral/inhaled therapy only) and 10 healthy volunteers matched for age and sex underwent MR-CPET. Exercise was performed on a MRI compatible ergometer (Lode, Groningen, The Netherlands) CPET data was collected using a commercial respiratory gas-analyser (Medgraphics, St. Paul, USA) with a modified MR compatible sampling umbilical.

Resting and peak exercise ventricular volumes and septal curvature were measured using a radial k-t SENSE real time sequence. Aortic flow was simultaneously continuously measured using a previously validated real-time UNFOLD-SENSE spiral PCMR sequence.

**Figure 1:**
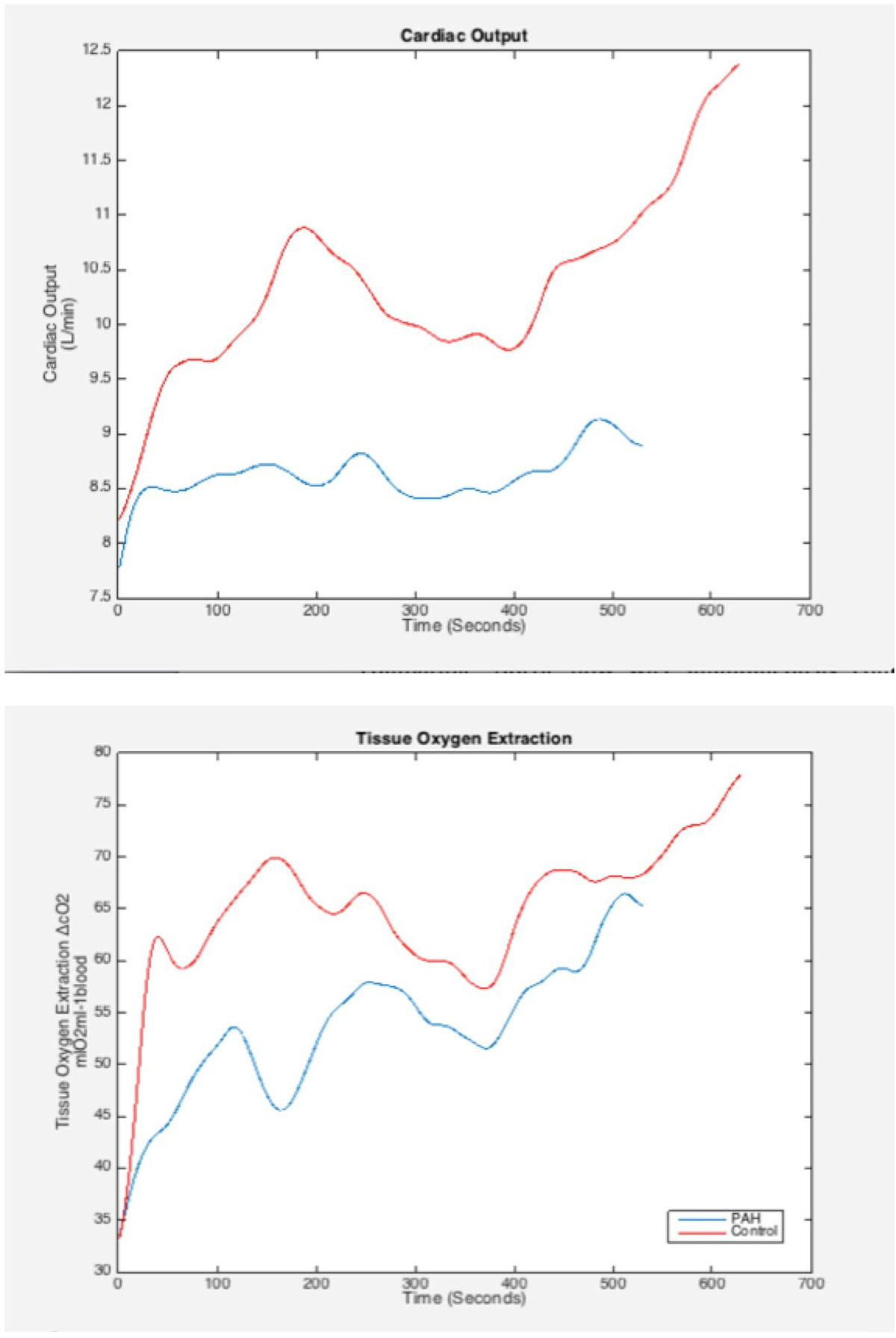
**a) Cardiac output b) Oxygen extraction**.

MR data was used to derive CO, HR and SV curves during exercise. Arteriovenous oxygen content gradient (AVO2) curves (a measure of tissue oxygen extraction) were calculated by dividing the VO2 and CO curves (Figure 1). Resting and peak exercise mean pulmonary artery pressures (mPAP) were estimated based on septal curvature using a previously validated technique.

Subjects also completed a six-minute walk test, experience survey and exercise history.

## Results

**Figure 2:**
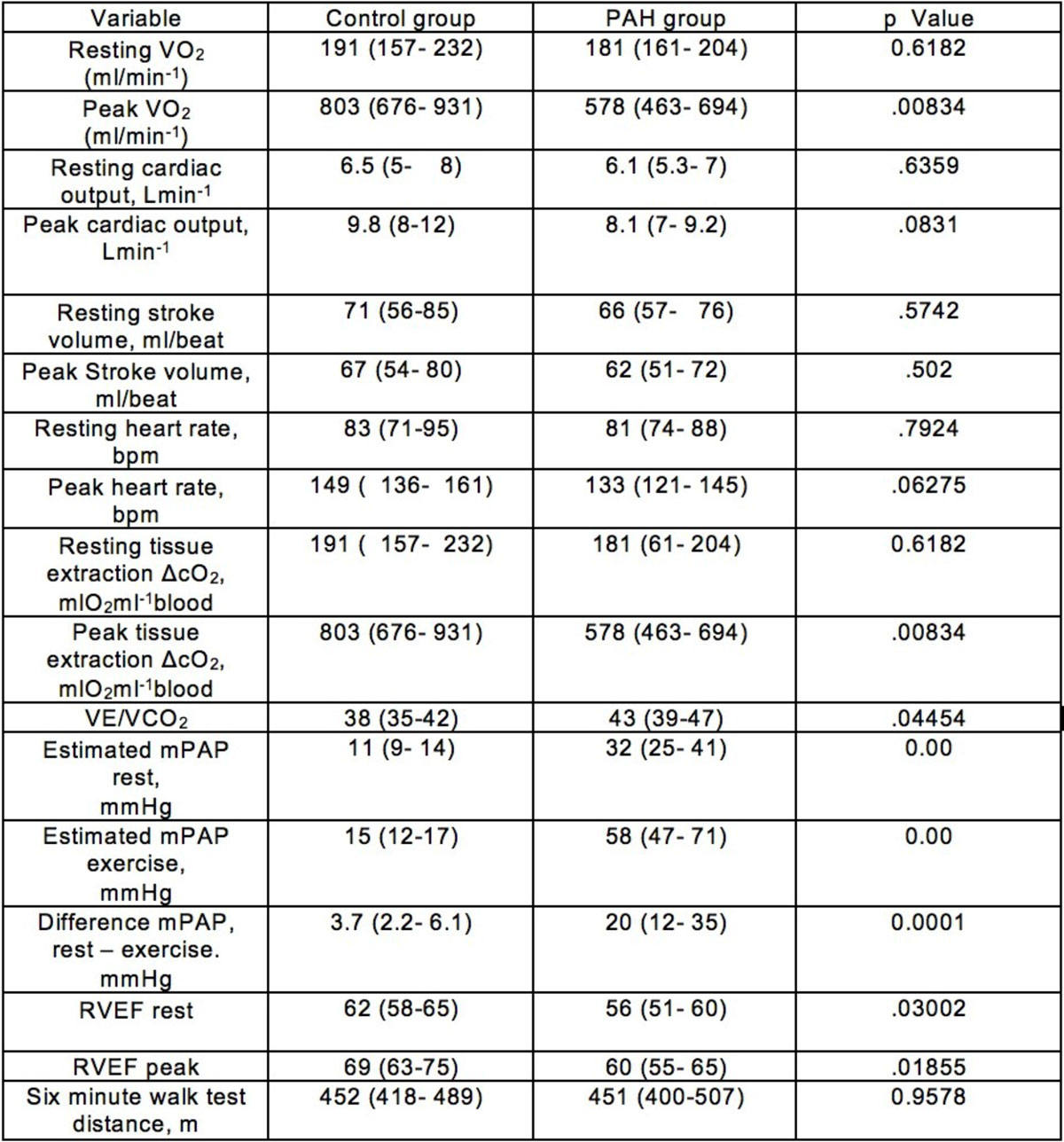
**MR-CPET findings**.

All children successfully completed the test without complication.Peak VO_2_ was significantly lower in the PAH group. This seems to be driven by both reduced tissue oxygen extraction and cardiac output although only reduced ΔcO_2_ was of statistical significance. MR-CPET measures at rest and exercise are shown in Figure 2.

RVEF was significantly lower in the PAH group both at rest and at peak exercise.There was a significant difference in the increase in estimated mPAP between the control and PAH groups. There was no significant difference in 6-minute walk test distance between the groups.

## Conclusions

MR-CPET is well tolerated in both healthy children and children with class I/II PAH. This study demonstrates that exercise intolerance in children with PAH is in part due to reduced tissue oxygen extraction and may open possibilities to study new pharmacological or exercise based therapeutic strategies.

